# Combined simultaneous FDG-PET/MRI with T1 and T2 mapping as an imaging biomarker for the diagnosis and prognosis of suspected cardiac sarcoidosis

**DOI:** 10.1186/s41824-021-00119-w

**Published:** 2021-12-16

**Authors:** Edward Cheung, Sarah Ahmad, Matthew Aitken, Rosanna Chan, Robert M. Iwanochko, Meyer Balter, Ur Metser, Patrick Veit-Haibach, Filio Billia, Yasbanoo Moayedi, Heather J. Ross, Kate Hanneman

**Affiliations:** 1 Department of Medical Imaging, Peter Munk Cardiac Centre, Toronto General Hospital, University of Toronto, 585 University Avenue, 1 PMB-298, Toronto, ON M5G 2N2 Canada; 2 Division of Cardiology, Peter Munk Cardiac Centre, University Health Network, University of Toronto, 585 University Ave, Toronto, ON M5G 2N2 Canada; 3 Division of Respiratory Medicine, Sinai Health System, University of Toronto, 600 University Ave, Toronto, ON M5G 1X5 Canada; 4 Division of Molecular Imaging, Department of Medical Imaging, University Health Network, University of Toronto, 585 University Avenue, Toronto, ON M5G 2N2 Canada

**Keywords:** Cardiac sarcoidosis, Sarcoidosis, FDG PET, Cardiac MRI, CMR, PET/MRI

## Abstract

**Purpose:**

To evaluate the diagnostic and prognostic significance of combined cardiac ^18^F-fluorodeoxyglucose (FDG) PET/MRI with T1/T2 mapping in the evaluation of suspected cardiac sarcoidosis.

**Methods:**

Patients with suspected cardiac sarcoidosis were prospectively enrolled for cardiac ^18^F-FDG PET/MRI, including late gadolinium enhancement (LGE) and T1/T2 mapping with calculation of extracellular volume (ECV). The final diagnosis of cardiac sarcoidosis was established using modified JMHW guidelines. Major adverse cardiac events (MACE) were assessed as a composite of cardiovascular death, ventricular tachyarrhythmia, bradyarrhythmia, cardiac transplantation or heart failure. Statistical analysis included Cox proportional hazard models.

**Results:**

Forty-two patients (53 ± 13 years, 67% male) were evaluated, 13 (31%) with a final diagnosis of cardiac sarcoidosis. Among patients with cardiac sarcoidosis, 100% of patients had at least one abnormality on PET/MRI: FDG uptake in 69%, LGE in 100%, elevated T1 and ECV in 100%, and elevated T2 in 46%. FDG uptake co-localized with LGE in 69% of patients with cardiac sarcoidosis compared to 24% of those without, *p* = 0.014. Diagnostic specificity for cardiac sarcoidosis was highest for FDG uptake (69%), elevated T2 (79%), and FDG uptake co-localizing with LGE (76%). Diagnostic sensitivity was highest for LGE, elevated T1 and ECV (100%). After median follow-up duration of 634 days, 13 patients experienced MACE. All patients who experienced MACE had LGE, elevated T1 and elevated ECV. FDG uptake (HR 14.7, *p* = 0.002), elevated T2 (HR 9.0, *p* = 0.002) and native T1 (HR 1.1 per 10 ms increase, *p* = 0.044) were significant predictors of MACE even after adjusting for left ventricular ejection fraction and immune suppression treatment. The presence of FDG uptake co-localizing with LGE had the highest diagnostic performance overall (AUC 0.73) and was the best predictor of MACE based on model goodness of fit (HR 14.9, *p* = 0.001).

**Conclusions:**

Combined cardiac FDG-PET/MRI with T1/T2 mapping provides complementary diagnostic information and predicts MACE in patients with suspected cardiac sarcoidosis.

## Introduction

Sarcoidosis is a multisystem disease characterized by development and accumulation of non-caseating granulomas in various organs. The most common sites of involvement are the lungs and thoracic lymph nodes (Iannuzzi et al. 2007). However, sarcoidosis can also involve the heart and other organs (Okada et al. 2018). Patients with cardiac sarcoidosis have a worse prognosis compared to patients with sarcoidosis without cardiac involvement (Birnie et al. 2016). Most deaths are due to ventricular arrhythmias, high-degree heart block, or heart failure resulting from progressive granulomatous infiltration or presence of myocardial fibrosis (Birnie et al. 2016; Grunewald et al. 2019).

Early identification of cardiac sarcoidosis is important given the risk of significant morbidity and mortality if untreated and the availability of therapeutic options. However, cardiac sarcoidosis remains a diagnostic challenge (Perry and Vuitch 1995; Rybicki et al. 1997). Endomyocardial biopsy remains the reference standard to confirm cardiac involvement, although the diagnostic yield is less than 25% (Vignaux 2005). Therefore, a positive biopsy result is definitive, whereas a negative result does not exclude this disease. Expert consensus criteria have also been proposed for diagnosing cardiac sarcoidosis, although these may also have limited diagnostic accuracy (Hulten et al. 2016). There is no reliable serum biomarker to confirm the diagnosis of cardiac sarcoidosis or assess response to therapy, and therefore clinicians are reliant on imaging for assessment of cardiac involvement and disease activity.

Advanced cardiac imaging techniques, including both cardiac magnetic resonance imaging (MRI) (Patel et al. 2009; Smedema et al. 2005) and ^18^F-labelled fluoro-2-deoxyglucose (FDG) positron emission tomography (PET) (Skali et al. 2013a; Youssef et al. 2012), are useful noninvasive tests for assessment of cardiac sarcoidosis. However, the best imaging modality for assessment of patients with suspected cardiac sarcoidosis has not yet been established (Gutberlet 2020). The recent development of integrated PET/MRI scanners might improve diagnosis and risk stratification of patients with cardiac sarcoidosis (Rischpler et al. 2013). Therefore, the objective of this study was to evaluate the diagnostic and prognostic significance of combined cardiac ^18^F-FDG PET/MRI in the evaluation of suspected cardiac sarcoidosis. We hypothesized that combined ^18^F-FDG PET/MRI with T1 and T2 mapping would provide complementary diagnostic information and predict development of future adverse cardiac events in patients with suspected cardiac sarcoidosis.

## Methods

This prospective cohort study was approved by the institutional research ethics board. Written informed consent was obtained from all participants. Between October 2016 and May 2021, consecutive patients with known or suspected cardiac sarcoidosis ≥ 18 years of age referred for clinically indicated PET/CT were recruited. Exclusion criteria included contraindication to PET/MRI, implanted cardiac pacemaker and defibrillator devices, and inability/unwillingness to follow the required diet instructions. Clinical data were obtained from patient history at the time of recruitment, telephone follow-up and the electronic patient record. Patients were classified as having a final diagnosis of cardiac sarcoidosis or not using the 2006 revised Japanese Ministry of Health and Welfare (JMHW) guideline as the reference standard, with exclusion of LGE as a minor criterion in order to evaluate the diagnostic accuracy of this imaging test in the study (Japanese Ministry Health and Welfare 2007).

### Primary and secondary outcomes

For evaluation of prognostic value, only new events from the time of PET/MRI were considered. The primary outcome of major adverse cardiac events (MACE) was defined as composite of cardiovascular death, aborted sudden cardiac death (either ventricular tachyarrhythmia leading to appropriate cardioverter defibrillator discharge or successfully resuscitated cardiac arrest), symptomatic ventricular tachyarrhythmia leading to cardioverter defibrillator implantation (ICD), symptomatic bradyarrhythmia leading to pacemaker implantation, cardiac transplantation, or hospitalization for decompensated heart failure. The secondary outcome of non-sustained ventricular tachycardia (NSVT) was defined as 3 or more consecutive beats arising below the atrioventricular node with an RR interval of > 100 beats/minute lasting < 30 s. Patients who did not experience an adverse event were censored at the time of their last clinical follow-up.

### PET/MRI acquisition

Patients were provided with detailed preparation instructions in order to suppress physiologic myocardial glucose uptake, including a high fat, high protein, low carbohydrate diet for the entire day before PET/MRI and a complete fast other than water for the immediate 12-h period prior to imaging (Williams and Kolodny 2008; Okumura et al. 2004). Adherence to the diet instructions was confirmed through direct questioning and evaluation of blood glucose. PET/MRI was performed 167 ± 38 min after intravenous administration of 426 ± 64 MBq of ^18^F-FDG using a 3 T scanner (Biograph mMR, Siemens Healthineers, Erlangen, Germany). The interval between injection and imaging was longer than typical clinical protocols due to an intervening clinical PET/CT scan. Blood glucose immediately prior to FDG injection was 5.3 ± 1.5 mmol/L. The cardiac MRI protocol included long-axis (2-, 3-, 4-chamber) and a stack of short-axis balanced cine steady-state free precession (SSFP) slices; T1 mapping using a modified Look-Locker Inversion Recovery (MOLLI) technique at basal, mid and apical short-axis locations, pre- and 15 min post-contrast (0.15 mmol/kg of gadolinium-based contrast agent, Gadobutrol, Bayer Healthcare); T2 mapping using a fast low-angle shot (FLASH) technique at matching short-axis locations pre-contrast; and phase-sensitive inversion recovery late gadolinium-enhanced (LGE) technique starting 12 min after contrast administration. Pixel-based T1 and T2 maps were automatically generated on the scanner with application of inline motion correction algorithms. List mode PET acquisition occurred simultaneously with the MRI examination in one bed position centered over the heart (10 min), with ECG gating and three-dimensional image reconstruction using ordered subset expectation maximization (three iterations, 21 subsets, point spread function, Gaussian filter, 4 mm FWHM). A 2-point Dixon scan was acquired for attenuation correction, and a four-compartment model attenuation map was calculated including air, fat, water and tissue.

### PET/MRI analysis

PET/MRI studies were analyzed blinded to all clinical information by two experienced fellowship trained cardiac imagers. PET and MRI images were fused by translating and rotating PET images onto the MRI coordinate system. PET/MRI findings were evaluated globally and according to the AHA 17-segment model (Cerqueira et al. 2002). Myocardial FDG uptake was categorized as none, diffuse, focal or focal-on-diffuse. Focal or focal-on-diffuse patterns of myocardial FDG uptake were considered positive, and none or diffuse FDG uptake were considered negative (Ohira et al. 2011). FDG uptake was quantified using cardiac metabolic volume (CMV), which was assessed as the volume of myocardium with a SUV intensity above a threshold ratio of 1.2 of left ventricular blood pool to cardiac maximum SUV (OsiriX MD v.12.0.1, Bernex, Switzerland) (Ahmadian et al. 2014). Ventricular volumes, function and mass were assessed as per established standards (Circle cmr42; Circle Cardiovascular Imaging, Calgary, Canada) (Schulz-Menger et al. 2020). Global and segmental LGE was evaluated visually. The predominant pattern of LGE was classified as subendocardial, mid-wall, subepicardial or transmural (Cummings et al. 2009). Global and segmental T1 and T2 relaxation times were assessed by contouring endocardial and epicardial borders on all short-axis images applying a 15% offset adjustment to eliminate in-plane partial volume artifacts. Extracellular volume (ECV) was calculated with input of pre- and post-contrast T1 and hematocrit (Robison et al. 2018). Maximum native T1, T2 and ECV were defined as the highest segmental value for each parameter, and the values were categorized as normal/abnormal based on established scanner-specific local reference ranges (elevated T2 > 45 ms; elevated T1 > 1286 ms; and elevated ECV > 30%). Myocardial inflammation was assessed using cardiac MRI findings by applying the updated Lake Louise Criteria (Ferreira et al. 2018). All PET/MRI images were evaluated for the presence of extra-cardiac disease including thoracic lymphadenopathy, pulmonary opacities and extra-cardiac thoracic FDG uptake typical in sarcoidosis (for example, in thoracic lymph nodes and in the lungs). Any abnormality on MRI was defined as LGE presence, elevated T2, elevated T1 and/or elevated ECV. Any abnormality on PET/MRI was defined as focal FDG uptake, LGE presence, elevated T2, elevated T1 and/or elevated ECV. PET/MRI findings were considered to co-localize when abnormalities were present in the same myocardial segment based on the AHA 17-segment model (for example, focal FDG uptake and LGE).

### Statistical analysis

Statistical analysis was performed using a commercially available software package, STATA v14.1 (StataCorp, College Station, Texas). A two-tailed *p* value of < 0.05 was considered statistically significant. All continuous data were tested for normal distribution using the Shapiro–Wilk test. Comparisons between groups were made by independent samples *t* test for continuous variables with normal distribution, Wilcoxon rank-sum test for continuous variables with non-normal distribution, and Fisher’s exact test for categorical variables. The diagnostic performance of PET/MRI findings was evaluated including sensitivity, specificity and area under the curve (AUC) using receiver operating characteristic analysis. In addition to evaluation of diagnostic test performance in the entire cohort, a sensitivity analysis was also performed, restricting the evaluation to the subgroup of patients not treated with immune suppression therapy prior to PET/MRI. Time-to-event survival analysis using Cox proportional hazard models was used to examine the association between PET/MRI findings and primary and secondary outcomes, both in univariable analysis and in multivariable analysis adjusting for left ventricular ejection fraction (LVEF) and anti-inflammatory therapy. Incremental value was assessed using nested models and the likelihood ratio test. Kaplan–Meier survival curves were calculated for visualization of cumulative event-free survival.

## Results

Forty-two patients (53 ± 13 years, 67% male) were included in this study (Table 1). The prevalence of cardiac sarcoidosis was 31% (*n* = 13). Twenty patients (48%) had biopsy-confirmed extra-cardiac sarcoidosis, and 6 (14%) were treated with immune suppression therapy at the time of PET/MRI (of whom 3 were positive on PET and 3 were negative on PET).

**Table 1:** Baseline clinical parameters

	All patients (*n* = 42)	Cardiac sarcoidosis (*n* = 13)	Not cardiac sarcoidosis (*n* = 29)	*P* value
Age, year	53 ± 13	59 ± 11	50 ± 14	0.039
Women, *n* (%)	14 (33%)	4 (31%)	10 (34%)	0.99
Body surface area, m^2^	2.0 ± 0.2	2.0 ± 0.2	2.0 ± 0.2	0.30
Systolic blood pressure, mmHg	124 ± 19	129 ± 23	121 ± 17	0.24
Diastolic blood pressure, mmHg	75 ± 10	79 ± 10	74 ± 10	0.20
Symptomatic, *n* (%)	24 (57%)	10 (77%)	14 (48%)	0.10
*Final diagnosis*				
Cardiac sarcoidosis	13 (31%)	13 (100%)	–	
Extra-cardiac sarcoidosis with no cardiac involvement	10 (24%)	–	10 (34%)	
Myocarditis	7 (17%)	–	7 (24%)	
Non-ischemic/inflammatory cardiomyopathy	12 (29%)	–	12 (41%)	
*Risk factors*				
Smoking history, *n* (%)	14 (33%)	7 (54%)	7 (24%)	0.08
Hypertension, *n* (%)	19 (45%)	7 (54%)	12 (41%)	0.52
Diabetes mellitus, *n* (%)	10 (24%)	2 (15%)	8 (28%)	0.47
Hypercholesterolemia, *n* (%)	18 (43%)	7 (54%)	11 (38%)	0.50
*Medications*				
Beta blocker, *n* (%)	22 (53%)	7 (54%)	15 (52%)	0.99
ACE inhibitor, *n* (%)	12 (29%)	4 (31%)	8 (28%)	0.99
ARB, *n* (%)	6 (14%)	2 (15%)	4 (14%)	0.99
Immune suppression therapy, *n* (%)	6 (14%)	2 (15%)	4 (14%)	0.99
Corticosteroids, *n* (%)	6 (14%)	2 (15%)	4 (14%)	0.99
Methotrexate, *n* (%)	2 (5%)	1 (8%)	1 (3%)	0.53

Variables are presented as mean ± standard deviation or number with percentage in parentheses. *P* values are for the comparison between patients with cardiac sarcoidosis and those without. Final diagnosis of cardiac sarcoidosis was established using modified 2006 revised Japanese Ministry of Health and Welfare (JMHW) guideline as the reference standard

*ACE* angiotensin-converting enzyme, *ARB* angiotensin II receptor blockers

### PET findings

FDG uptake on PET was positive in 9/13 (69%) patients with cardiac sarcoidosis compared to 9/29 (31%) without (Table 2). Among patients without cardiac sarcoidosis with focal FDG uptake, the final diagnosis was myocarditis in 2 and non-ischemic or other inflammatory cardiomyopathy in 7. The pattern of FDG uptake was focal in 17 patients (40%) and focal on diffuse in one patient (2%). Only one patient (2%) without cardiac sarcoidosis had diffuse uptake likely due to inadequate physiologic myocardial glucose suppression. CMV and number of FDG-positive segments were significantly higher in patients with cardiac sarcoidosis compared to those without (median 39 cm^3^ vs. 16 cm^3^, *p* = 0.019 and median 11 vs. 5 segments, *p* = 0.031, respectively). The most common segments with focal FDG uptake were the basal inferolateral wall (38% for patients with and without cardiac sarcoidosis), basal anterior septum (38% in patients with cardiac sarcoidosis and 28% in those without) and mid-inferolateral wall (23% in patients with cardiac sarcoidosis and 28% in those without).

**Table 2:** Combined PET/MRI findings

	All patients (*n* = 42)	Cardiac sarcoidosis (*n* = 13)	Not cardiac sarcoidosis (*n* = 29)	*P* value
Cardiac MRI				
*Left ventricle*				
LVEDVi, ml/m^2^	89 ± 26	88 ± 18	90 ± 29	0.84
LVEF, %	52 ± 11	52 ± 12	52 ± 10	0.97
LVMi, g/m^2^	50 ± 12	50 ± 13	51 ± 14	0.86
*Tissue characterization*				
LGE presence, *n* (%)	33 (79%)	13 (100%)	20 (69%)	0.038
LGE extent, number of segments	4.7 ± 4.8	4.2 ± 2.4	4.9 ± 5.6	0.49
Global native T1, ms	1263 ± 73	1274 ± 74	1257 ± 73	0.49
Regional native T1, ms	1344 ± 91	1377 ± 58	1328 ± 100	0.040
Elevated native T1, *n* (%)	30 (73%)	13 (100%)	17 (61%)	0.008
Global ECV, %	28 ± 6	28 ± 6	27 ± 6	0.73
Regional ECV, %	34 ± 8	38 ± 8	33 ± 8	0.044
Elevated ECV, *n* (%)	30 (73%)	13 (100%)	17 (61%)	0.008
Global native T2, ms	40 ± 3	41 ± 2	39 ± 3	0.09
Regional native T2, ms	45 ± 5	46 ± 5	44 ± 5	0.045
Elevated native T2, *n* (%)	12 (29%)	6 (46%)	6 (21%)	0.15
MRI positive, *n* (%)	33 (79%)	13 (100%)	20 (69%)	0.038
*Extra-cardiac findings*				
Pulmonary opacities, *n* (%)	18 (43%)	10 (77%)	8 (28%)	0.006
Thoracic lymphadenopathy, *n* (%)	16 (38%)	9 (69%)	7 (24%)	0.014
*FDG-PET*				
FDG-PET positive, *n* (%)	18 (43%)	9 (69%)	9 (31%)	0.041
Focal pattern of uptake, *n* (%)	17 (40%)	8 (61%)	9 (31%)	0.09
Focal on diffuse pattern of uptake, *n* (%)	1 (2%)	1 (7%)	0 (0%)	0.31
Diffuse FDG uptake, *n* (%)	1 (2%)	0 (0%)	1 (3%)	0.99
FDG-PET extent, number of segments	2.4 ± 4.2	3.7 ± 4.9	1.9 ± 4.2	0.035
CMV, cm^3^	23 ± 43	39 ± 43	15 ± 41	0.015
Extra-cardiac FDG uptake typical of sarcoidosis, *n* (%)	17 (40%)	8 (62%)	9 (31%)	0.09
*Combined PET/MRI*				
PET+ and/or MRI+, *n* (%)	34 (81%)	13 (100%)	21 (72%)	0.043
PET−/MRI−, *n* (%)	8 (19%)	0 (0%)	8 (28%)	0.043
PET+/MRI−, *n* (%)	1 (2%)	0 (0%)	1 (3%)	0.99
PET−/MRI+, *n* (%)	16 (38%)	4 (31%)	12 (41%)	0.73
PET+/MRI+, *n* (%)	17 (40%)	9 (69%)	8 (27%)	0.017
Co-localized focal FDG uptake and LGE, *n* (%)	16 (38%)	9 (69%)	7 (24%)	0.014
Co-localized focal FDG uptake and high T1, *n* (%)	16 (38%)	9 (69%)	7 (24%)	0.014
Co-localized focal FDG uptake and high T2, *n* (%)	11 (26%)	6 (46%)	5 (17%)	0.07

Variables are presented as mean ± standard deviation or number with percentage in parentheses. *P* values are for the comparison between patients with cardiac sarcoidosis and those without

PET was considered positive (+) if the pattern of FDG uptake was focal or focal on diffuse. MRI was considered positive (+) if LGE was present or if native T1, T2 or ECV were elevated

*LV* Left ventricle, *LVEDVi* indexed left ventricular end-diastolic volume, *LVESVi* indexed left ventricular end-systolic volume, *LVEF* left ventricular ejection fraction, *LVCO* left ventricular cardiac output, *LVMi* indexed left ventricular mass, *RV* right ventricle, *RVEDVi* indexed right ventricular end-diastolic volume, *RVESVi* indexed right ventricular end-systolic volume, *RVEF* right ventricular ejection fraction, *RVCO* right ventricular cardiac output, *LGE* late gadolinium enhancement, *ECV* extracellular volume, *MRI* magnetic resonance imaging, *FDG* fluorodeoxyglucose, *PET* positron emission tomography

### Cardiac MRI findings

Overall, 79% of patients had at least one MRI abnormality (100% of those with cardiac sarcoidosis vs. 69% of those without). There was no significant difference in left ventricular size, function or mass between groups. Patients with cardiac sarcoidosis had higher prevalence of LGE and higher maximum segmental T1, T2 and ECV compared to those without. Maximum segmental native T1 and ECV were elevated in all patients with LGE. The pattern of LGE in patients with cardiac sarcoidosis was most commonly mid-wall (85% vs. 41% in patients without cardiac sarcoidosis). No patient had a pattern of LGE consistent with myocardial infarction. The most common segments with LGE were the basal anterior septum (92% in patients with cardiac sarcoidosis and 34% in those without) and basal inferolateral wall (61% in patients with cardiac sarcoidosis and 34% in those without). All patients with elevated T2 had corresponding LGE and elevated T1/ECV and met updated Lake Louise criteria for myocardial inflammation (Ferreira et al. 2018).

### Combined PET/MRI findings

**Fig. 1:**
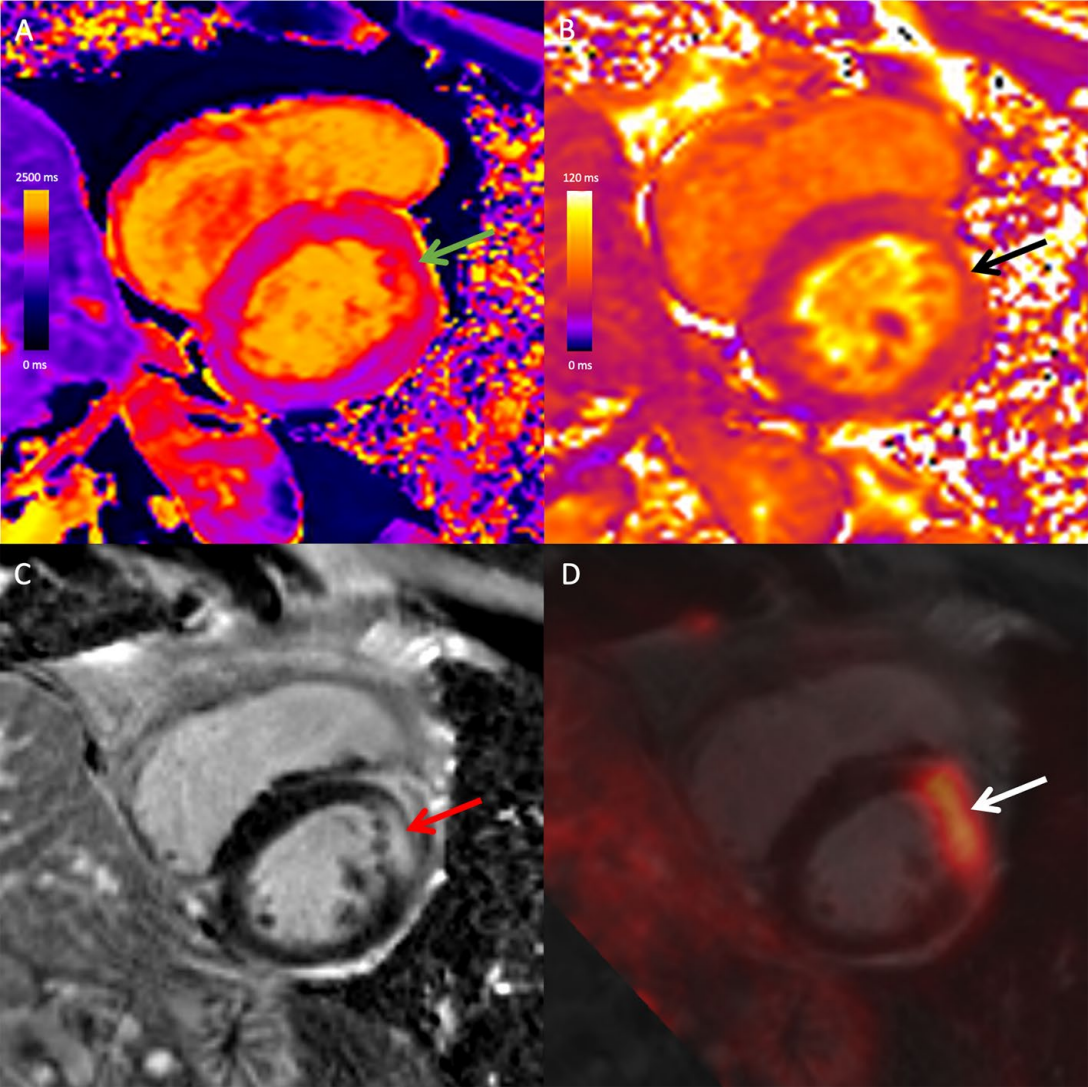
Combined 18F-FDG PET/MRI images in a 67-year-old male with cardiac and extra-cardiac sarcoidosis. Mid-ventricular short-axis native T1 map (**A**), native T2 map (**B**), late gadolinium-enhanced (LGE) image (**C**), and fused 18F-FDG PET and LGE image (**D**) demonstrate high T1 (green arrow), high T2 (black arrow), mid-wall LGE (red arrow) and co-localizing focal FDG uptake (white arrow) at the mid-anterior wall. There was extra-cardiac FDG uptake in mediastinal and hilar lymph nodes (not shown). The study was positive on both PET and MRI, likely reflecting active cardiac sarcoidosis with myocardial inflammation

**Fig. 2:**
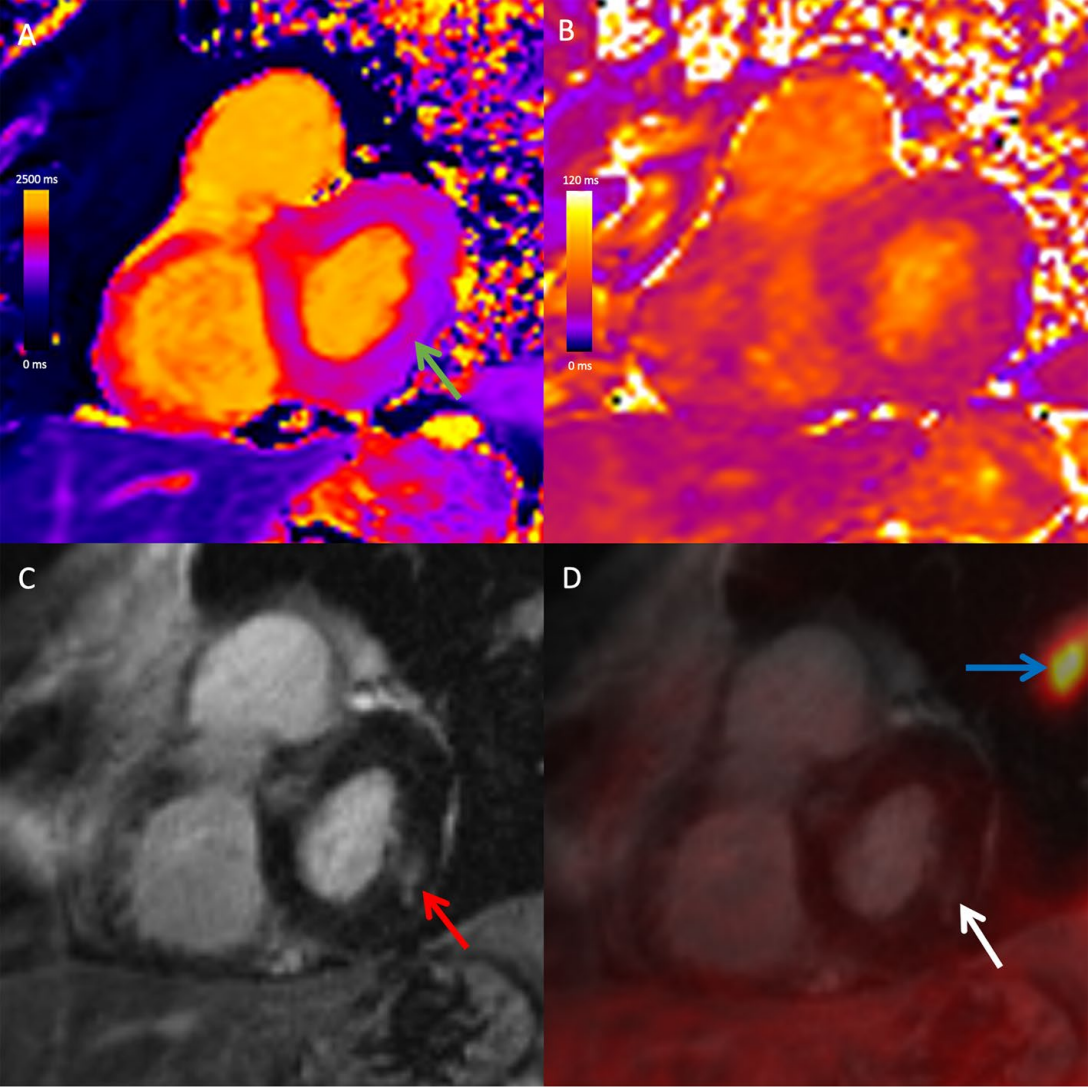
Combined 18F-FDG PET/MRI images in a 72-year-old male with cardiac and extra-cardiac sarcoidosis. Basal short-axis native T1 map (**A**), native T2 map (**B**), late gadolinium-enhanced (LGE) image (**C**), and fused 18F-FDG PET and LGE image (**D**) demonstrate slightly elevated native T1 (green arrow) and corresponding mid-wall LGE (red arrow) at the basal inferolateral wall. There was no corresponding elevation of native T2 or focal FDG uptake (white arrow). There was extra-cardiac FDG uptake in mediastinal and hilar lymph nodes (not shown) and in the lung parenchyma (blue arrow) in keeping with extra-cardiac sarcoidosis. With respect to the heart, the study was negative on PET and positive on MRI, likely reflecting chronic, burnt-out cardiac sarcoidosis

Overall, 81% of patients had at least one abnormality on combined PET/MRI (100% in patients with cardiac sarcoidosis vs. 72% in those without, *p* = 0.043). Among patients with cardiac sarcoidosis, 4 patients (31%) were negative on PET but positive on MRI and 9 patients (69%) were positive on both PET and MRI (Figs. 1 and 2). No patient with cardiac sarcoidosis was negative on both PET and MRI or positive on PET but negative on MRI.

**Fig. 3:**
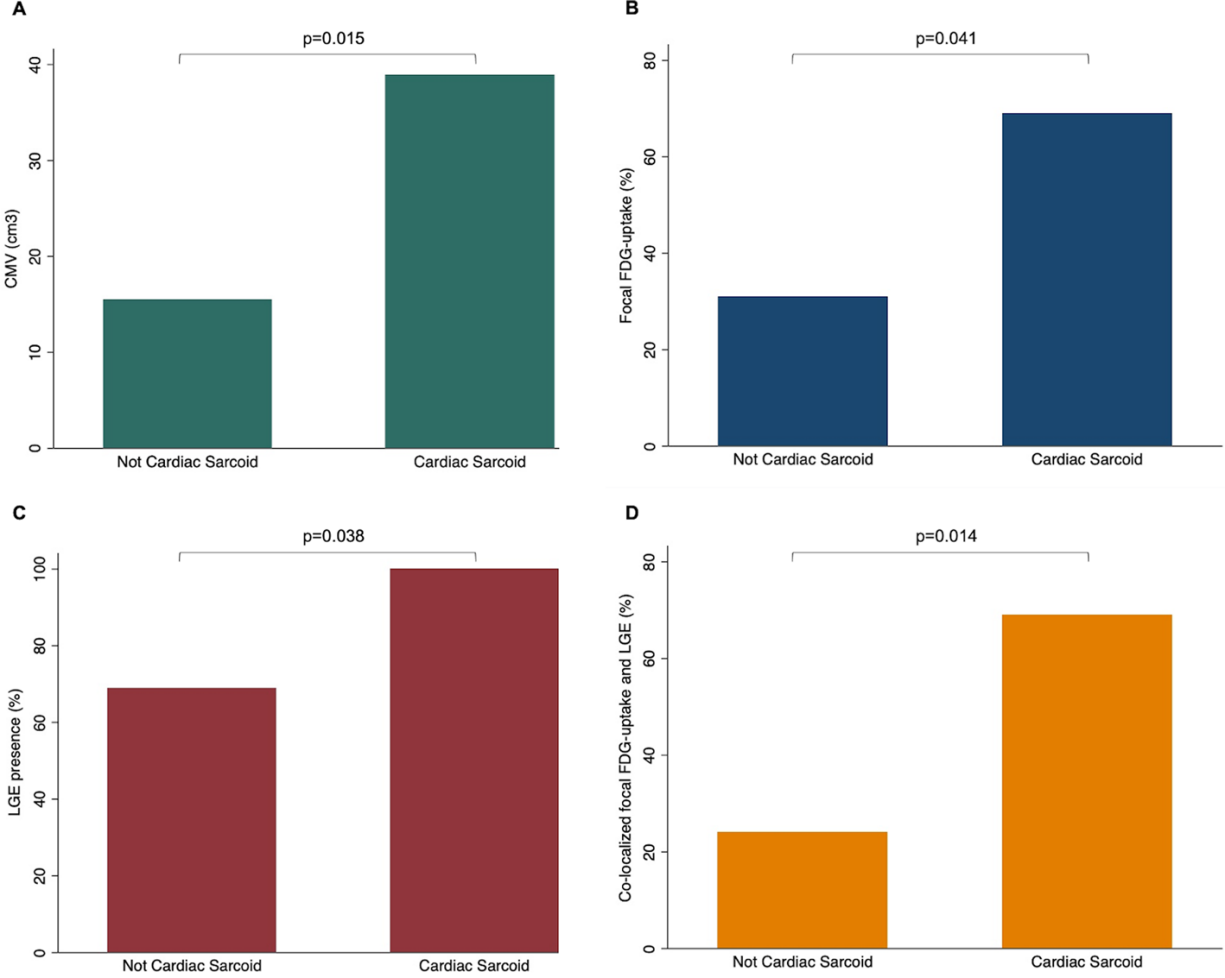
Bar charts for PET and MRI findings by patient group. CMV (**A**), focal FDG uptake (**B**), LGE presence (**C**) and co-localized FDG uptake and LGE presence (**D**)

Focal FDG uptake co-localized with LGE and with elevated T1/ECV in at least one myocardial segment in 69% of patients with cardiac sarcoidosis (vs. 24% of those without cardiac sarcoidosis, *p* = 0.014) and co-localized with elevated T2 in 46% patients with cardiac sarcoidosis (vs. 17% of those without cardiac sarcoidosis, *p* = 0.07) (Fig. 3). Focal FDG uptake was present but did not co-localize with LGE in two patients (5%, both without cardiac sarcoidosis) and did not co-localize with elevated T2 in 7 patients (14%, 3 with cardiac sarcoidosis and 4 without).

### Diagnostic accuracy

In the entire cohort, diagnostic specificity for cardiac sarcoidosis was highest for focal FDG uptake (69%), elevated T2 (79%), co-localizing focal FDG uptake with LGE or with elevated T1 (76%), and co-localizing focal FDG uptake with elevated T2 (83%) (Table 3). Specificity was significantly higher for co-localizing focal FDG uptake and elevated T2 compared to focal FDG uptake alone (*p* = 0.045). Diagnostic sensitivity was highest for LGE presence, elevated T1 and elevated ECV (all with 100% sensitivity but low specificity). Overall diagnostic performance was highest for focal FDG uptake co-localizing with LGE or with elevated T1.

**Table 3:** Diagnostic accuracy of PET/MRI findings for cardiac sarcoidosis

	Sensitivity (95% CI)	Specificity (95% CI)	Positive predictive value (95% CI)	Negative predictive value (95% CI)	Area under the curve (95% CI)
*All patients (n* = *42)*					
FDG-PET positive (PET+)	69 (39, 91)	69 (49, 85)	50 (26, 74)	83 (63, 95)	0.69 (0.54, 0.85)
LGE presence	100 (75, 100)	31 (15, 51)	39 (23, 58)	100 (66, 100)	0.66 (0.66, 0.74)
High native T1	100 (75, 100)	39 (22, 59)	43 (26, 63)	100 (72, 100)	0.70 (0.60, 0.79)
High ECV	100 (75, 100)	39 (22, 59)	43 (26, 63)	100 (72, 100)	0.70 (0.60, 0.79)
High native T2	46 (19, 75)	79 (59, 92)	50 (21, 79)	76 (57, 90)	0.62 (0.46, 0.78)
Updated Lake Louise criteria	46 (19, 75)	79 (59, 92)	50 (21, 79)	76 (57, 90)	0.62 (0.46, 0.78)
MRI positive (MRI+)	100 (75, 100)	31 (15, 51)	39 (23, 58)	100 (66, 100)	0.66 (0.66, 0.74)
Combined PET/MRI (PET+ and/or MRI+)	100 (75, 100)	28 (13, 47)	38 (22, 56)	100 (63, 100)	0.64 (0.56, 0.72)
Co-localized focal FDG uptake and LGE	69 (39, 91)	76 (57, 90)	56 (30, 80)	85 (65, 96)	0.73 (0.57, 0.88)
Co-localized focal FDG uptake and high T1	69 (39, 91)	76 (57, 90)	56 (30, 80)	85 (65, 96)	0.73 (0.57, 0.88)
Co-localized focal FDG uptake and high T2	46 (19, 75)	83 (64, 94)	55 (23, 83)	77 (59, 90)	0.65 (0.49, 0.80)
*Patients not treated with immune suppression therapy (n* = *36)*					
FDG-PET positive (PET+)	73 (39, 94)	72 (51, 88)	53 (27, 79)	86 (64, 97)	0.72 (0.56, 0.89)
LGE presence	100 (72, 100)	32 (15, 54)	39 (22, 59)	100 (63, 100)	0.66 (0.57, 0.75)
High native T1	100 (72, 100)	38 (15, 54)	39 (22, 59)	100 (63, 100)	0.66 (0.57, 0.75)
High ECV	100 (72, 100)	38 (15, 54)	39 (22, 59)	100 (63, 100)	0.66 (0.57, 0.75)
High native T2	46 (17, 77)	75 (53, 90)	46 (17, 77)	75 (53, 90)	0.60 (0.42, 0.78)
Updated Lake Louise criteria	46 (17, 77)	75 (53, 90)	46 (17, 77)	75 (53, 90)	0.60 (0.42, 0.78)
MRI positive (MRI+)	100 (72, 100)	24 (9, 45)	37 (20, 56)	100 (54, 100)	0.62 (0.54, 0.71)
Combined PET/MRI (PET+ and/or MRI+)	100 (72, 100)	28 (12, 49)	38 (21, 58)	100 (59, 100)	0.64 (0.55, 0.73)
Co-localized focal FDG uptake and LGE	73 (39, 94)	76 (55, 91)	57 (29, 82)	86 (65, 97)	0.74 (0.58, 0.91)
Co-localized focal FDG uptake and high T1	73 (39, 94)	76 (55, 91)	57 (29, 82)	86 (65, 97)	0.74 (0.58, 0.91)
Co-localized focal FDG uptake and high T2	46 (17, 77)	80 (59, 93)	50 (19, 81)	77 (56, 91)	0.63 (0.45, 0.80)

95% confidence intervals (CI) are in parentheses. Final diagnosis of cardiac sarcoidosis was established using modified 2006 revised Japanese Ministry of Health and Welfare (JMHW) guideline as the reference standard

*PET* Positron emission tomography, *LGE* late gadolinium enhancement, *ECV* extracellular volume, *MRI* magnetic resonance imaging, *ROC* receiver operating characteristic

PET was considered positive (+) if the pattern of FDG uptake was focal or focal on diffuse. MRI was considered positive (+) if LGE was present or if native T1, T2 or ECV were elevated

In the subset of patients not treated with immune suppressive therapy, sensitivity, negative predictive value and AUC were slightly higher for several PET/MRI parameters, compared with analysis of the entire cohort.

### Prognostic value

After median follow-up duration of 634 ± 380 days, 13 patients experienced MACE (incidence rate 22%/year, non-mutually exclusive events: 3 cardiac deaths, 1 aborted sudden cardiac death, 8 symptomatic ventricular tachyarrhythmias leading to ICD placement, 1 cardiac transplantation and 2 hospitalization for heart failure) and 12 patients experienced NSVT (incidence rate 20%/year).

**Fig. 4:**
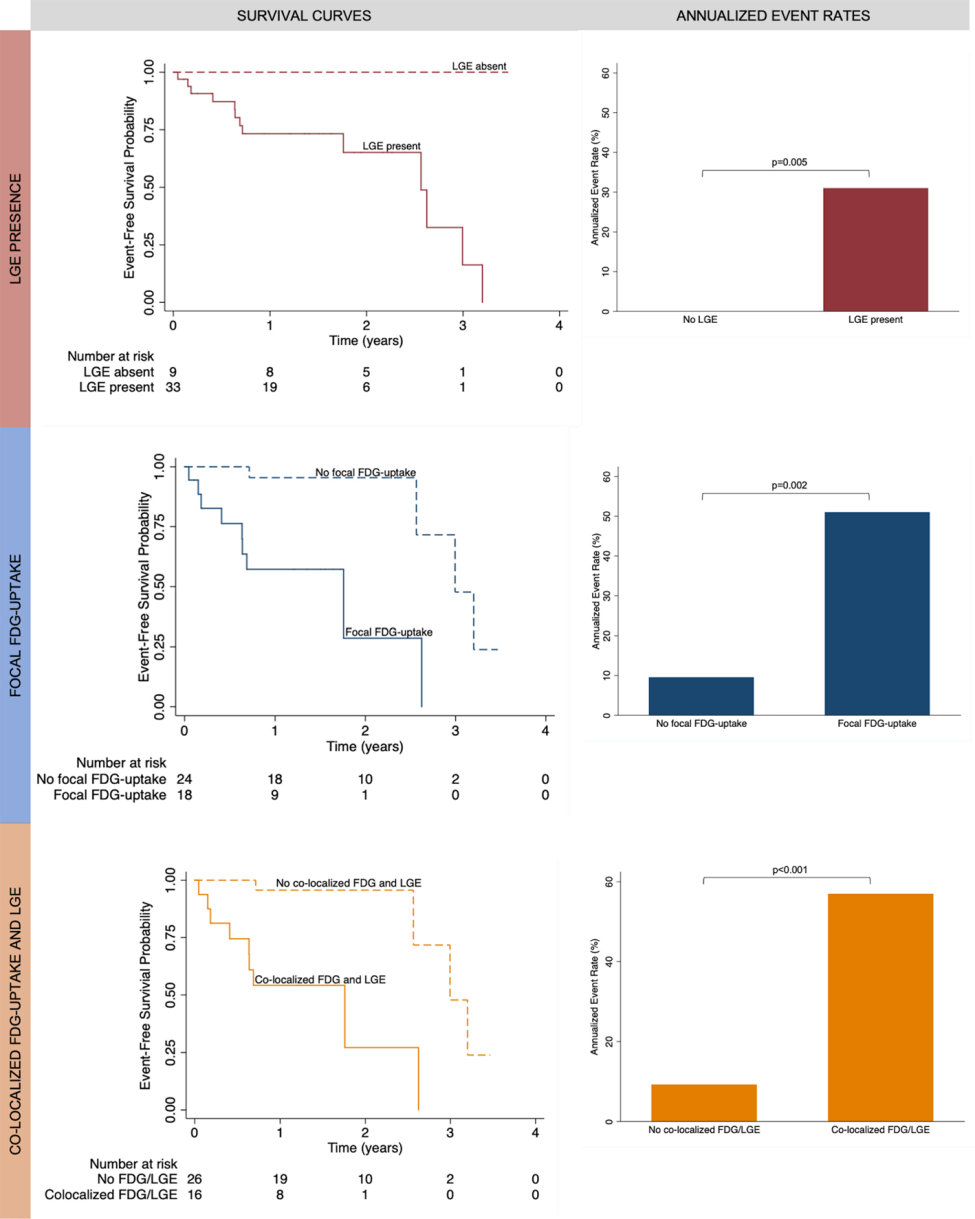
Kaplan–Meier survival probability curves for the primary end-point of major adverse cardiac events (MACE) demonstrate that patients with late gadolinium enhancement (LGE), focal FDG uptake, and co-localized focal FDG uptake and LGE have worse event-free survival compared to patients without those findings. The number of patients at risk is shown at the bottom of the figures. Annualized event rates were higher in patients with LGE, focal FDG uptake, and co-localized focal FDG uptake and LGE

Patients with focal FDG uptake on PET had 11 times higher risk of MACE compared to those without (HR 10.8, *p* = 0.003) (Table 4). The risk increased with the extent of FDG uptake, with a one segment increase in FDG uptake associated with a 23% increase in the risk of MACE. Annualized MACE rates were significantly higher in patients with positive FDG uptake (51%) compared to those without FDG uptake (10%), *p* = 0.002 (Fig. 4).

**Table 4:** Univariable and multivariable time-to-event survival analysis for primary and secondary end-points

	Univariable			Multivariable		
	HR (95% CI)	*P* value	AIC	HR (95% CI)	*P* value	AIC
*MACE*						
Focal FDG uptake	10.8 (2.3, 51.2)	0.003	62	14.7 (2.8, 78.4)	0.002	56
Focal FDG uptake (number of segments)	1.2 (1.1, 1.4)	< 0.001	61	1.2 (1.1, 1.3)	0.004	62
CMV (10 cm^3^ increase)	1.2 (1.0, 1.3)	0.022	70	1.3 (1.1, 1.5)	0.006	64
Native T1 (100 ms increase)	2.2 (1.3, 3.6)	0.003	66	1.8 (1.0, 3.1)	0.044	66
ECV (1% increase)	1.1 (1.0, 1.1)	0.003	66	1.0 (0.99, 1.1)	0.07	67
Native T2 (10 ms increase)	3.9 (1.5, 10.2)	0.005	67	2.8 (1.0, 7.5)	0.041	66
Elevated T2	8.2 (2.2, 31.3)	0.002	63	9.0 (2.2, 37.7)	0.002	59
LVEF	0.93 (0.89, 0.98)	0.008	67	0.93 (0.88, 0.98)	0.008	69
Co-localized focal FDG uptake and LGE	11.8 (2.5, 55.6)	0.002	60	14.9 (2.8, 78.4)	0.001	56
Co-localized focal FDG uptake and high T1	11.8 (2.5, 55.6)	0.002	60	14.9 (2.8, 78.4)	0.001	56
Co-localized focal FDG uptake and high T2	6.0 (1.8, 20.8)	0.004	66	7.4 (2.0, 27.0)	0.003	61
*NSVT*						
Focal FDG uptake	9.5 (2.0, 46.2)	0.005	57	12.0 (2.3, 63.1)	0.003	52
Focal FDG uptake (number of segments)	1.2 (1.1, 1.4)	< 0.001	54	1.2 (1.0, 1.3)	0.005	56
CMV (10 cm^3^ increase)	1.2 (1.0, 1.4)	0.026	63	1.3 (1.1, 1.5)	0.009	57
Native T1 (10 ms increase)	1.1 (1.0, 1.1)	0.003	59	1.1 (1.0, 1.1)	0.06	60
ECV (1% increase)	1.1 (1.0, 1.2)	0.002	58	1.1 (1.0, 1.1)	0.05	59
Native T2 (1 ms increase)	1.1 (1.0, 1.3)	0.008	61	1.1 (1.0, 1.2)	0.07	61
Elevated T2	7.1 (1.8, 27.8)	0.005	58	7.2 (1.7, 30.0)	0.007	55
LVEF	0.93 (0.88, 0.97)	0.007	59	0.93 (0.88, 0.98)	0.007	62
Co-localized focal FDG uptake and LGE	10.6 (2.2, 50.9)	0.003	56	12.3 (2.3, 63.0)	0.002	52
Co-localized focal FDG uptake and high T1	10.6 (2.2, 50.9)	0.003	56	12.3 (2.3, 63.0)	0.002	52
Co-localized focal FDG uptake and high T2	5.2 (1.5, 18.6)	0.011	61	7.2 (1.7, 30.0)	0.007	55

All patients who experienced MACE had LGE, elevated native T1 and elevated ECV. Annualized MACE rates dichotomized by the presence and absence of LGE were 31% and 0%, respectively. Patients with elevated T2 had 8 times higher risk of MACE compared to those without (HR 8.2, *p* = 0.002). A 10 ms increase in native T1 was associated with an 8% increase in the risk of MACE, a 1 ms increase in native T2 was associated with a 14% increase in the risk of MACE, and a 1% increase in ECV was associated with an 8% increase in the risk of MACE.

In nested models with focal FDG uptake, model fit for MACE was improved by the addition of LGE extent (*χ*^2^(*df* = 1) = 12.2, *p* < 0.001), indicating that LGE extent on MRI has independent and incremental value in predicting the risk of MACE beyond FDG uptake on PET. The presence of focal FDG uptake co-localizing with LGE or with elevated T1 was associated with 12 times higher risk of MACE compared to patients without and was the best predictor of MACE among the imaging findings evaluated based on model goodness of fit. Annualized MACE rates were significantly higher in patients with co-localizing FDG uptake and LGE (57%) compared to those without FDG uptake (9%), *p* < 0.001.

In multivariable analysis, combined PET/MRI findings remained as significant predictors of MACE even after adjusting for LVEF and anti-inflammatory therapy, including FDG uptake (HR 14.7, *p* = 0.002), elevated T2 (HR 9.0, *p* = 0.002) and FDG uptake co-localizing with LGE (HR 14.9, *p* = 0.001).

## Discussion

Cardiac involvement in patients with sarcoidosis is associated with poor prognosis. However, early identification and risk stratification are challenging due to non-specific clinical manifestations and patchy myocardial involvement which can limit the sensitivity of diagnostic tests including endomyocardial biopsy. We have shown that FDG-PET/MRI provides complementary information in patients with suspected cardiac sarcoidosis, combining the strengths of both imaging modalities in a single comprehensive study that allows for co-registration of accurate and strongly prognostic imaging data. Key strengths of each imaging modality include characterization of myocardial tissue and quantification of ventricular function on MRI and assessment of metabolic processes such as inflammation on PET (Sanchez Tijmes et al. 2021). Focal FDG uptake on PET and elevated T2 on MRI had the highest diagnostic specificity among the individual imaging parameters evaluated and are most useful in ruling in cardiac sarcoidosis when present. Cardiac MRI tissue characterization findings including LGE, elevated T1 and elevated ECV had 100% sensitivity and are therefore useful in ruling out cardiac sarcoidosis when absent. Overall diagnostic performance was highest for focal FDG uptake that co-localized with LGE or with elevated T1, highlighting the complementary information provided by combined PET/MRI compared to either modality alone. Cardiac PET/MRI findings also have strong prognostic value, particularly when evaluated together as a combined modality. The presence of focal FDG uptake co-localizing with LGE was the best predictor of MACE among the imaging findings evaluated.

In cardiac sarcoidosis, segments that are positive for LGE on MRI but negative for focal FDG uptake on PET likely reflect myocardial scarring/fibrosis in the setting of chronic disease without active inflammation. Compared to LGE, focal FDG uptake on PET is a more specific marker of active disease reflecting metabolically active immune cells and typically resolves or regresses following anti-inflammatory treatment (Coulden et al. 2020). FDG-PET is particularly well suited for investigation of cardiac sarcoidosis due to the presence of macrophage-dense regions in areas of active sarcoidosis (Li et al. 2020). Determining the acuity of cardiac involvement in patients with cardiac sarcoidosis is imperative, as data suggest that steroid or other anti-inflammatory therapy should be administered to patients with active inflammation before left ventricular systolic function declines (Skali et al. 2013b). Identification of chronic cardiac involvement, even without active inflammation, is also important for risk stratification, as fibrosis is strongly associated with adverse cardiac events including ventricular arrhythmias (Flamée et al. 2020). Patients with extensive myocardial scar by LGE or elevated T1/ECV may warrant closer follow-up and consideration of prophylactic ICD implantation (Al-Khatib et al. 2018).

Only a few prior studies have investigated the performance of combined PET/MRI in cardiac sarcoidosis, with varying imaging and analysis approaches (Hanneman et al. 2017; Dweck et al. 2017; Wicks et al. 2018). To our knowledge, this study is the first to describe the diagnostic and prognostic value of combined cardiac PET/MRI in patients with suspected cardiac sarcoidosis with an imaging protocol that includes T1 and T2 mapping. Our results are concordant with the study by Wicks et al., which found that combined abnormalities on both PET and MRI were the strongest predictor of MACE compared to the absence of any imaging abnormalities or singular abnormalities on either PET or MRI (Wicks et al. 2018). However, in their study, 22% of patients had PET abnormalities with no MRI abnormalities, while in our study no patients with cardiac sarcoidosis were positive on PET but negative on MRI. This could be related to differences in the classification of PET abnormalities between studies, as they included diffuse patterns of FDG uptake in their analysis. Diffuse FDG uptake can be seen in the setting of severe diffuse active cardiac inflammation but is more commonly seen in the context of inadequate dietary preparation (Ohira et al. 2011). In our study, only a single patient had diffuse FDG uptake which likely reflected physiologic myocardial glucose uptake as all MRI parameters were normal (including T2) and the patient subsequently endorsed incomplete adherence to the diet instructions. The proportion of patients with extra-cardiac disease was lower in our study compared to others which is likely related to inclusion criteria as we included patients with suspected isolated cardiac sarcoidosis in addition to those with known extra-cardiac disease (Hanneman et al. 2017; Dweck et al. 2017; Wicks et al. 2018).

Diagnostic specificity was higher for all imaging parameters evaluated when our analysis was restricted to the subgroup of patients with known extra-cardiac sarcoidosis. This highlights the fact that diagnostic accuracy is dependent on the patient sample evaluated and is expected to be higher when patients with more severe disease (such as patients with advanced, biopsy-proven sarcoidosis) are compared to a control group with no disease (such as patients with extra-cardiac sarcoidosis with no evidence of cardiac disease). We included a consecutive sample of patients with suspected cardiac sarcoidosis who had a range of disease severity in our study, which accounts for more modest diagnostic performance compared to other studies that evaluated patients with cardiac sarcoidosis compared to healthy controls (Puntmann et al. 2017) or restricted study inclusion to only patients with biopsy-proven disease (Smedema et al. 2005). Importantly, FDG uptake is not specific to cardiac sarcoidosis and can be seen in the setting of other causes of myocardial inflammation including myocarditis (Hanneman et al. 2017; Chen and Jeudy 2019).

There is conflicting information on the prognostic significance of FDG uptake in cardiac sarcoidosis in the literature to date, with some studies demonstrating an association between FDG abnormalities and adverse cardiac events (Blankstein et al. 2014), while other studies have not (Wicks et al. 2018; Mehta et al. 2008). Both the presence and extent of FDG uptake were associated with MACE and NSVT in our cohort of patients with suspected cardiac sarcoidosis. Lack of a significant association of PET abnormalities with adverse events in prior studies could be related to differences in the classification and analysis of PET findings and shorter follow-up duration (Wicks et al. 2018; Mehta et al. 2008). We also found that cardiac MRI findings including LGE, native T1, native T2, ECV and LVEF were associated with MACE and NSVT. All patients who experienced an adverse cardiac event had LGE, elevated native T1 and elevated ECV. Our results are consistent with a prior meta-analysis that found that LGE was a strong predictor of adverse events in cardiac sarcoidosis, with a relative risk of cardiovascular mortality of 10.7 (Hulten et al. 2016). Myocardial fibrosis/scar secondary to inflammation from sarcoid granulomas is thought to be the dominant substrate for arrhythmias, including ventricular tachycardia, in cardiac sarcoidosis (Kumar et al. 2015). In our study, FDG uptake co-localizing with LGE or with elevated T1 was the best predictor of MACE and NSVT. MRI findings had incremental prognostic value compared to PET findings alone, highlighting the strength of combined imaging for prognostic evaluation in this clinically complex disease.

The results of this study have several clinical implications. When available, combined PET/MRI with T1/T2 mapping could improve diagnostic performance and risk stratification of patients with suspected cardiac sarcoidosis compared to either modality alone. FDG uptake on PET is particularly useful to rule in active disease when positive and may also be an important noninvasive biomarker for monitoring response to anti-inflammatory therapy (Coulden et al. 2020). Cardiac MRI findings are more sensitive and are therefore helpful in ruling out cardiac sarcoidosis when negative. Combined PET/MRI including T2 mapping may also be useful in the setting of diffuse FDG uptake to distinguish between physiologic FDG uptake in the setting of inadequate diet preparation (in which case T2 would be expected to be normal) and diffuse myocardial inflammation (in which case T2 would be expected to be elevated). Given that elevated native T1 was concordant with the presence of LGE, this technique may be particularly useful in patients with a contraindication to administration of gadolinium-based contrast which is required for LGE imaging. Combined PET/MRI findings are associated with adverse cardiac event risk, and therefore detection of these imaging biomarkers could impact patient management by supporting an indication for prophylactic defibrillator implantation or escalation of medical therapy.

Our study has several limitations including lack of a biopsy reference standard for cardiac sarcoidosis, lack of perfusion imaging and a modest sample size. We used modified JMHW criteria as the reference standard in our study, which has been utilized in most diagnostic accuracy studies to date, although these criteria have not been validated against any other standard. It is possible that select patients could have been misclassified, particularly those that did not meet JMHW criteria but had evidence of focal FDG uptake on PET. Although cardiac sarcoidosis is relatively rare, the number of patients included is modest. FDG uptake on PET was not interpreted in the context of perfusion imaging, which could potentially account for the more modest diagnostic performance of PET compared to prior PET/CT studies. However, the focus of this study was on combined PET/MRI findings. The interval between FDG injection and imaging was longer than many clinical protocols which could impact PET imaging findings (Slart et al. 2021). However, compared to oncology indications, a longer interval is acceptable for imaging of inflammation and has been previously demonstrated not to impact PET results in cardiac sarcoidosis (Hanneman et al. 2017). Finally, our evaluation focused on the left ventricular myocardium. Evaluation of the diagnostic and prognostic value of right ventricular abnormalities on PET/MRI could be performed in a separate study. All imaging was performed at a single tertiary referral hospital, and therefore results may not be generalizable to all centers given that PET/MRI scanners are not widely available.

In conclusion, combined cardiac FDG PET/MRI with T1 and T2 mapping provides complementary diagnostic and prognostic information in patients with suspected cardiac sarcoidosis. These data support the necessity for future large, longitudinal follow-up studies to evaluate the value of PET/MRI in assessing response to therapy and to evaluate the independent and incremental prognostic value of imaging findings over clinical parameters.

## Data Availability

Not available for sharing.

## Abbreviations

AUC: Area under curve
ECV: Extracellular volume
FDG: Fluorodeoxyglucose
LGE: Late gadolinium enhancement
HR: Hazard ratio
ICD: Implantable cardioverter defibrillator
JMHW: Japanese Ministry of Health and Welfare
MACE: Major adverse cardiac events
NSVT: Non-sustained ventricular tachycardia
PET: Positron emission tomography
